# Amplification of cell signaling and disease resistance by an immunity receptor Ve1Ve2 heterocomplex in plants

**DOI:** 10.1038/s42003-022-03439-0

**Published:** 2022-05-25

**Authors:** Melanie Kalischuk, Boje Müller, Adriana F. Fusaro, Champa P. Wijekoon, Peter M. Waterhouse, Dirk Prüfer, Lawrence Kawchuk

**Affiliations:** 1 Department of Agriculture and Agri-Food Canada, Lethbridge, AB T1J 4B1 Canada; 2 School of Life and Environmental Sciences, University of Sydney, Sydney, NSW 2006 Australia; 3 Department of Plant Agriculture, University of Guelph, Guelph, ON N1G 2W1 Canada; 4 Fraunhofer Institute for Molecular Biology and Applied Ecology IME, Schlossplatz 8, 48143 Münster, Germany; 5 Institute of Medical Biochemistry, Federal University of Rio de Janeiro (UFRJ), Rio de Janeiro, 21941-590 Brazil; 6 Canadian Centre for Agri-Food Research in Health and Medicine, 351 Taché Avenue, R2020, Winnipeg, MB R2H 2A6 Canada; 7 School of Earth, Environmental and Biological sciences, Queensland University of Technology, Brisbane, QLD 4001 Australia; 8 Institute of Plant Biology and Biotechnology, University of Münster, Schlossplatz 8, 48143 Münster, Germany

**Keywords:** Pattern recognition receptors in plants, Plant signalling

## Abstract

Immunity cell-surface receptors Ve1 and Ve2 protect against fungi of the genus *Verticillium* causing early dying, a worldwide disease in many crops. Characterization of microbe-associated molecular pattern immunity receptors has advanced our understanding of disease resistance but signal amplification remains elusive. Here, we report that transgenic plants expressing Ve1 and Ve2 together, reduced pathogen titres by a further 90% compared to plants expressing only Ve1 or Ve2. Confocal and immunoprecipitation confirm that the two receptors associate to form heteromeric complexes in the absence of the ligand and positively regulate signaling. Bioassays show that the Ve1Ve2 complex activates race-specific amplified immunity to the pathogen through a rapid burst of reactive oxygen species (ROS). These results indicate a mechanism by which the composition of a cell-surface receptor heterocomplex may be optimized to increase immunity against devastating plant diseases.

## Abstract (web summary)

Transgenic plants expressing both Ve1 and Ve2 conferred enhanced signaling and disease resistance in susceptible potato in a race-specific manner, a step forward in generating disease resistant plants against Verticillium.

## Introduction

Plants recognize hostile microbes by detecting molecular patterns within pathogen avirulence (*Avr*) effectors. These microbe-associated molecular pattern (MAMP) receptors confer innate immunity by promoting the formation of cell surface complexes that initiate signal transduction in the host, followed by attenuation^1,2^. Various pattern-recognition receptors (PRRs) for MAMPs have been characterized in plants, including receptor-like proteins (RLPs) containing leucine-rich repeats, such as Ve^3^, Cf^4^, LeEIX^5^, and ELR^6^ and receptor-like kinases (RLKs) such as FLS2^7^ and Xa21^8^. However, few cell surface receptors have been identified that confer innate immunity to pathogens of agriculturally important crops, and our understanding of the associated signal transduction mechanisms to improve performance is limited.

There is growing evidence that PRRs form regulatory complexes to induce innate immunity responses. For example, the FLS2 kinase detects a conserved peptide in the bacterial flagellin protein (flg22) and forms a ligand-dependent complex with the receptor kinase BRASSINOSTEROID INSENSITIVE 1 ASSOCIATED KINASE 1 (BAK1) that initiates the signals that confer innate immunity before internalization by endocytosis^9^. Immunoprecipitation and structural data have confirmed that FLS2 and BAK1 form a ligand-dependent complex within minutes in the presence of flagellin. Among the RLPs, Cf-2 forms complexes that determine pathogen race specificity^10^ and LeEIX dimerization regulates signal attenuation^11^. An associative mechanism has also been described for the damage-associated endogenous peptide receptors (PEPR1 and PEPR2), which form signaling-competent complexes with BAK1 for ligand-dependent internalization^12,13^. Many RLPs constitutively interact with RLK SUPPRESSOR OF BIR1-1 (SOBIR1) and ligand binding leads to the recruitment of BAK1or related SOMATIC EMBRYOGENESIS RECEPTOR KINASEs (SERKs) as co-receptors that activate signaling^14^. Characterization of the Arabidopsis RLP23 receptor for necrosis- and ethylene-inducing-like proteins (NLPs) of the necrotrophic fungal pathogen *Botrytis cinerea* suggested a pre-invasive resistance^15^, and results with RLP42 showed a rapid evolution of fungal endopolygalacturonase (PGs) sensors with distinct pattern specificities in closely related Brassicae^16^. As pathogen effectors may evolve rapidly to produce new races that circumvent recognition by PRRs and thus overcome resistance, characterization of ligand perception and subsequent signal transduction and attenuation is important for the successful introduction of durable genetic disease resistance.

In tomato (*Solanum lycopersicon*), the closely-linked inverted genes *Ve1* and *Ve2* encode homologous RLPs that prevent premature death triggered by fungi of the genus *Verticillium* in more than 200 plant species worldwide, including many important crops^17^. Vascular wilt diseases such as verticillium wilt that cause water restriction are increasingly problematic in food production with climate change and impending water shortages in many areas of the world. Since the initial description of verticillium wilt resistance in tomato, the *Ve* genes have been bred into many commercial tomato varieties^18^. The tomato Ve proteins include an N-terminal signal peptide, leucine-rich repeats with putative glycosylation sites, a hydrophobic membrane-spanning domain, and a short cytoplasmic C-terminus containing a dileucine E/DxxxLL (Ve1) or tyrosine YxxΦ1 (Ve2) motif as putative endocytosis signals^3^. The tomato LeEix2 protein is a cell surface receptor for the fungal ethylene-inducing elicitor xylanase, and requires a C-terminal tyrosine to induce the hypersensitive response^19^. Evidence of PRR endocytosis in plants includes the constitutive internalization of these receptors through a series of events including dimerization and the formation of clathrin pits^20,21^. Many RLPs and RLKs are physically clustered and appear to regulate signal activation and attenuation as receptors or co-receptors, although the biological function of several remains to be elucidated^22^.

Several tomato genes are required for innate immunity including *Enhanced Disease Susceptibility 1* (*EDS1*), which enables resistance to a wide range of pathogens including Ve-mediated resistance^23^. The suppression of tomato *Ve1, BAK1*, *Mek2* (MAP kinase kinase), *Nrc1* (hypersensitive response-associated cell death), *Acif1* (F-box protein), and *Ndr1* (non-race-specific disease resistance) by virus-induced gene silencing (VIGS) also compromised resistance to *V. dahliae*^24^. The *Ve1* transcript is present at low levels in non-infected plants but is induced in plants infected by *Verticillium* spp., whereas the *Ve*2 transcript is constitutively expressed^25^. Both Ve1 and Ve2 interact with SOBIR1, which may function as a scaffold protein to stabilize receptor complexes^26,27^. Immunity receptor associations have also been further characterized by direct labeling, allowing the spatiotemporal analysis of complex formation and dissolution^9,11,26–28^.

To gain insight into the signal transduction pathways triggered by Ve proteins and the role of cell surface complexes in this process, we expressed tagged Ve proteins individually and together in transgenic plants to compare native sequences and variants lacking the putative endocytosis signals E/DxxxLL and YxxΦ1. Mutant receptors lacking these motifs were incapable of undergoing ligand-induced internalization but this had no impact on the perception of MAMPs, complex formation or signal transduction, leading to race-specific disease resistance. Furthermore, the formation of pathogen-independent Ve1Ve2 heteromeric complexes stimulated receptor-induced co-localization and achieved a remarkable 90% reduction in race-specific pathogen titres compared to the individual receptors.

## Results

### Plant immunity to *Verticillium* is increased by expressing Ve1 and Ve2 together

**Fig. 1:**
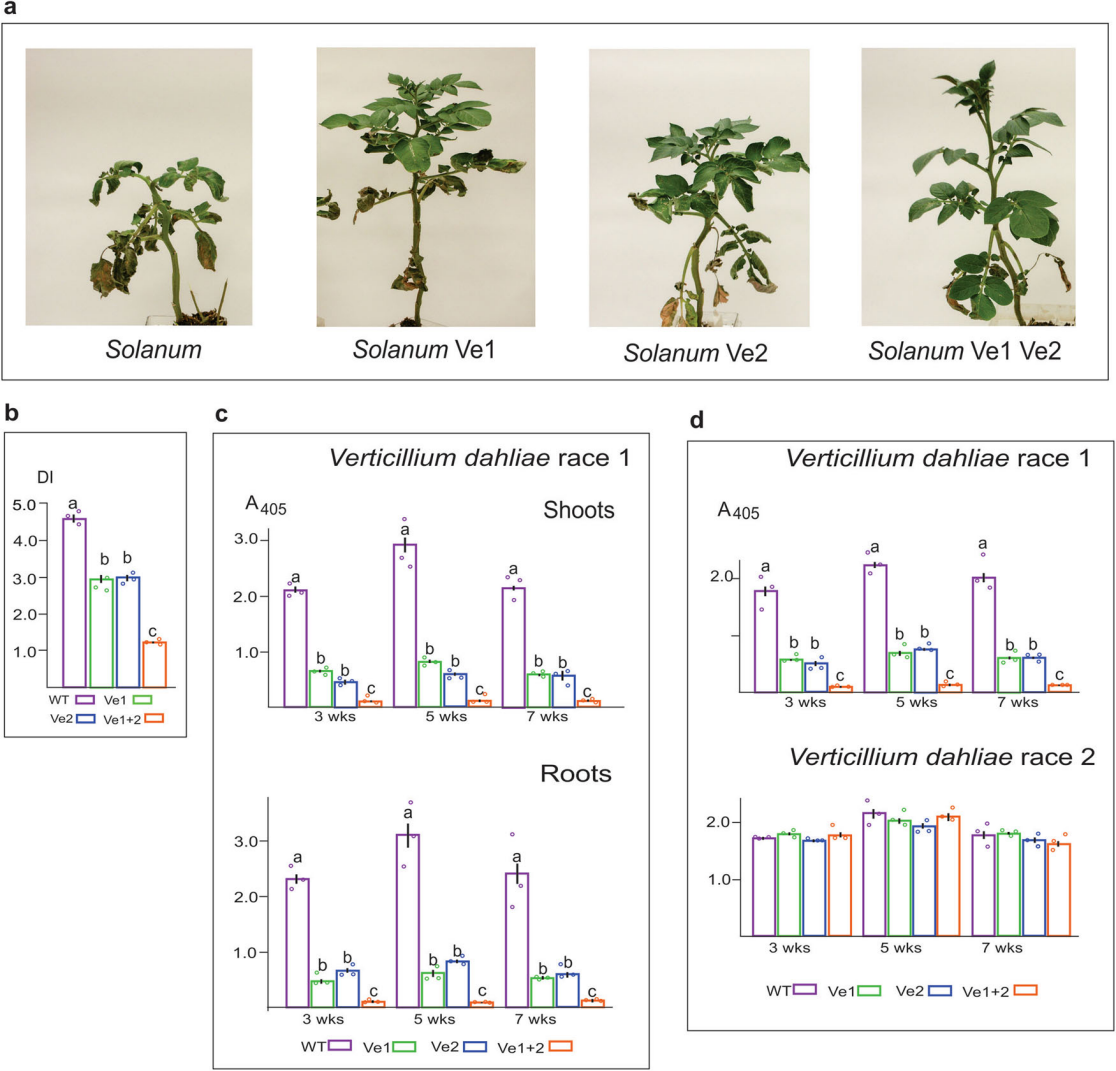
Plant innate immunity levels in stably transformed *Solanum tuberosum*. **a** Disease wilt and necrosis five weeks after inoculation with an aggressive isolate of *Verticillium albo-atrum* race 1. Suspensions of 10^7^/ml conidia were introduced into the wounded roots of 5 week old tissue culture potato plantlets stably transformed with each of the native *Ve* genes transcribed by the CaMV35S promoter. Images are representative plants from three independent experiments. Disease symptoms progress from the soil to the apex with wilting, chlorosis, and necrosis of leaves proceeding leaf loss. **b** Disease index (DI) means and standard error of the means were determined for 50 plants of each genotype, rated 10 weeks postinoculation with *V. albo-atrum* race 1, according to the percentage of tissue visually showing symptoms from three (*n*) independent experiments. Significance was determined using one-way ANOVA followed by Turkey-Kramer multiple comparisons (*p* < 0.001) and indicated by different letters for each time point. **c** Pathogen titers were determined 3, 5, and 7 weeks postinoculation with *V. dahliae* in roots and shoots of each potato plant using double antibody serological (DAS) enzyme linked immunosorbent assay (ELISA) and absorbance determined at a wavelength of 405 nm. Means and standard error of the means were determined six inoculated plants from three (*n*) independently replicated experiments. A significant reduction in pathogen titre (*p* < 0.001) was observed in those tissues transformed with Ve1 and Ve2, reducing pathogen titers to 2% of that detected in the controls. **d** Plants of stably transformed potato were also inoculated with either *Verticillium dahliae* race 1 isolate or race 2 isolate. Pathogen titers were determined in shoots of plants at 3, 5, and 7 weeks postinoculation with DAS ELISA. Results show that there was no immunity to race 2 of the pathogen even in the plants possessing the Ve1 and Ve2.

To characterize the innate immunity conferred by Ve1 and Ve2, we expressed either *Ve*1 or *Ve*2 under the control of the strong and constitutive Cauliflower Mosaic Virus 35 S promoter in the susceptible potato variety *Desiree* with each of the genes individually and carried out reciprocal crosses to stack both genes in the same line (Fig. 1, Supplementary Fig. 1). Before infection, the transformed plants and their progeny phenotypically resembled the wild-type parental lines, indicating that Ve1 and/or Ve2 showed no detrimental effect under normal conditions (Supplementary Fig. 2). Subsequent analyses of transgenic lines inoculated with aggressive race 1 isolates of *Verticillium albo-atrum* (Fig. 1a–c) showed that disease resistance produced less wilting, chlorosis, necrosis, and eliminated premature death (Fig. 1a), but also revealed a significant increase in disease resistance in plants expressing both *Ve*1 and *Ve*2 (Fig. 1b). Pathogen titers were determined by ELISA in the roots and shoots of the infected potato plants, confirming that the phenotypic responses represented resistance rather than tolerance (Fig. 1c)^3,29,30^. Hence in the plants transformed with single *Ve*1 or *Ve*2 genes, the pathogen titers were 25% of wild-type controls, and were further reduced by 90% in transgenic lines expressing both receptors simultaneously (Fig. 1c).

We also inoculated the potato plants with race 1 or race 2 isolates of *Verticillium dahliae* (Fig. 1d). The results with *V. dahliae* race 1 were similar to those reported above for *V. albo-atrum* race 1, with individual Ve genes reducing pathogen titres and both genes together producing only 2% of the pathogen compared to wild-type controls (Fig. 1d top). Interestingly, there was no significant difference between any of the transgenic lines and wild-type Desiree controls in response to *V. dahliae* race 2 (Fig. 1d bottom), indicating that neither *Ve1* nor *Ve2*, alone or together, conferred resistance to this race of the pathogen and that race-specificity was retained with amplified immunity (Fig. 1c).

### Epitope tagging of the Ve proteins facilitates tracking by confocal microscopy

**Fig. 2:**
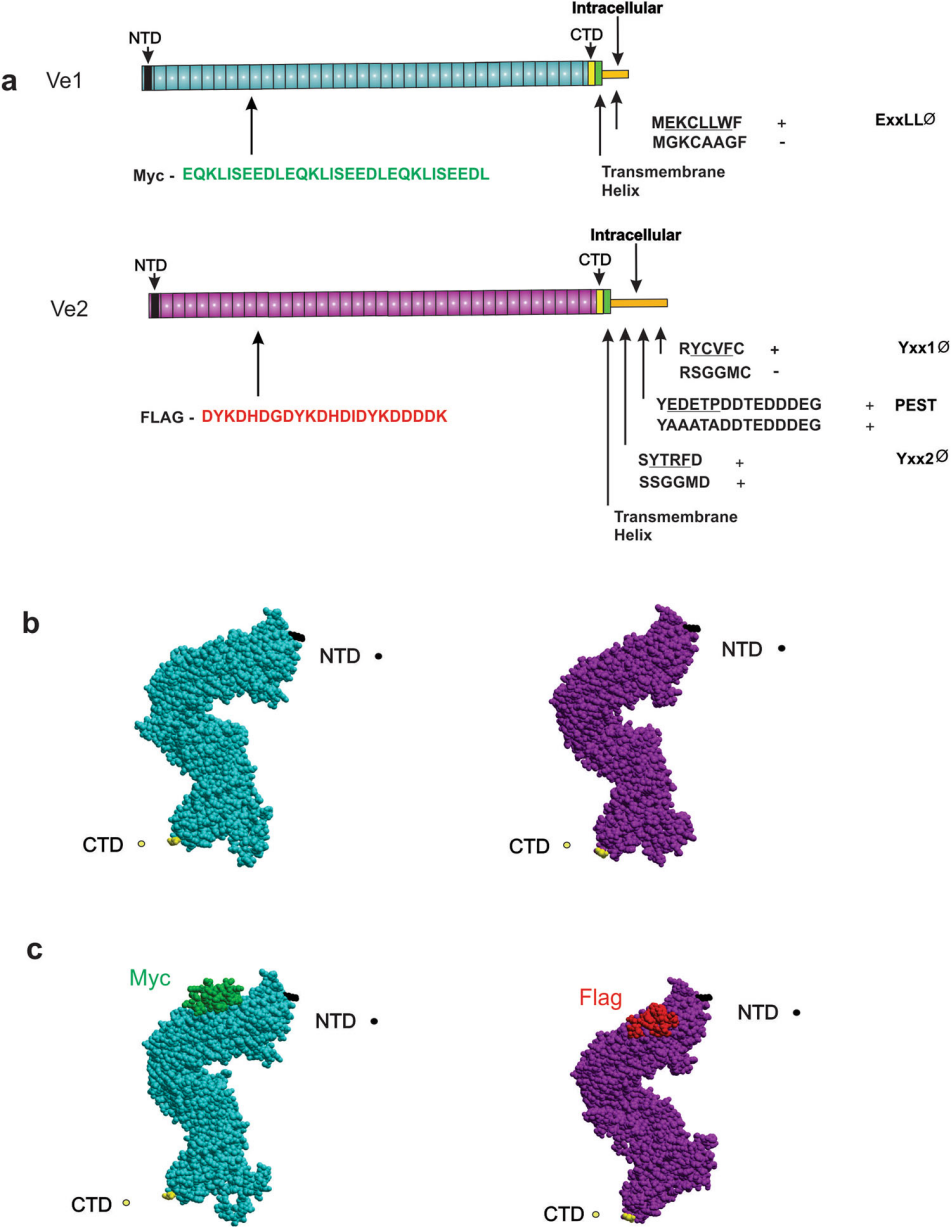
Signal positions and predicted secondary structure of Ve receptors. **a** Insertion sites of the myc-Ve1 (green) and FLAG-Ve2 (red) are shown in a series of modified receptors in relation to N-terminal (black), the hydrophobic plasma membrane spanning (green), and intracellular (gold) region with the putative C-terminal (yellow) signals. Influence of residue changes are indicated (+ or -) for Ve1E/DXXXLΦ, Ve2YXXΦ1, Ve2YXXΦ2, Ve2PEST and surrounding sequences. **b** Model of the Ve receptor ectodomains based on the GSO1 protein kinase (Ve1 blue and Ve2 purple) showing the proximity of the N-terminal (black Ve1 K38 and Ve2 K36) and C-terminal (yellow Ve1 N983 and Ve2 V982) domains. Models were generated with MolSoft (San Diego, CA). **c** Locations of the inserted Ve1 Myc (green) and Ve2 FLAG (red) epitopes and impact on the modelled receptor ectodomain.

The much greater resistance only conferred by simultaneous expression of both Ve receptors suggested the formation of complexes. To test this hypothesis, we created tagged versions of both receptors by incorporating unique epitopes, to facilitate independent tracking of expression and localization. The triple-Myc epitope was inserted by site-directed mutagenesis into Ve1 and the FLAG epitope into Ve2, in both cases selecting the leucine-rich repeat region as the target site (Fig. 2a, Supplementary Fig. 1)^9,26^. The precise insert positions at Ve1K237 and Ve2K235 were selected to minimize the impact on overall secondary/tertiary structure, to avoid the terminal endocytosis motifs, and to ensure epitope accessibility^31^. Predicted structures of the Ve proteins resembled the crystal structures of Toll-like receptors (TLRs), FLS2, and GSO1 formed by multiple leucine-rich repeats capped at each end by other domains^32–34^. Extracellular leucine-rich repeats in the Ve and FLS2 proteins represent a significant portion of the receptors, placing only the C terminus in proximity of the cell membrane (Fig. 2b). The Myc and FLAG epitopes are not predicted to disrupt this structure, but both epitopes are exposed for immunodetection (Fig. 2c). To determine the functionality of specific residues within the putative endocytosis signals of Ve1 and Ve2, we prepared a series of amino acid substitutions in the C-terminal dileucine (E/DxxxLΦ) motif of Ve1 and the C-terminal tyrosine-like motifs (YxxΦ) of Ve2 by site-directed mutagenesis (Fig. 2a, Supplementary Fig. 1).

### Immunity conferred by *Ve* genes does not require ligand-dependent endocytosis

**Fig. 3:**
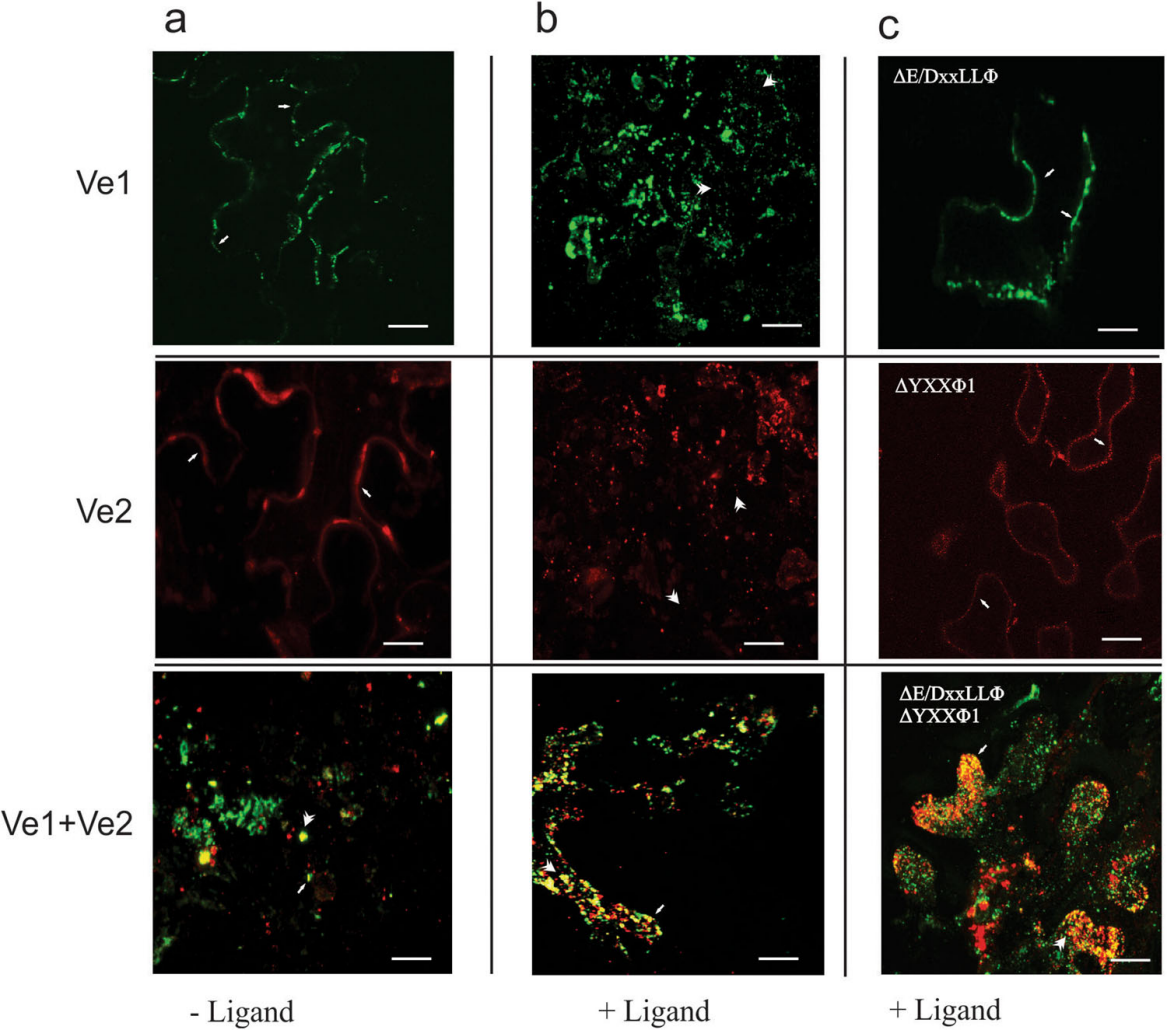
Cellular localization of the tagged Ve receptors and receptors lacking the pathogen dependent endocytosis signals. Confocal z-stack imaging microscopy of transient *Nicotiana benthamiana* bioassays 5 days postinoculation. **a** In the absence of *Verticillium dahliae* race 1 pathogen ligand (left column), the Ve receptors accumulate at the cell surface (arrows), except when both Ve1 (green) and Ve2 (red) receptors are present (left column bottom row). Image indicates associations between the Ve receptors (yellow) and the occurrence of heteromer endocytosis. **b** Addition of ligand (middle column) causes both Ve1 and Ve2 individual receptors to be internalized (arrowheads) through ligand dependent endocytosis. **c** Addition of ligand does not induce endocytosis in the Ve receptors lacking the Ve1E/DxxxLΦ or Ve2YxxΦ1 motifs (right column). Elimination of the endocytosis signals also does not impede heteromer endocytosis in cells containing both Ve receptors (bottom right). Confocal differential interference contrast (DIC) bright field microscopy is shown in Fig. S3.

The behavior of the tagged constructs was monitored by fluorescence microscopy in the leaves of *Nicotiana benthamiana* plants infiltrated with *Agrobacterium tumefaciens* strains carrying *Ve1* or *Ve2*, or co-infiltrated with both strains, followed by the staining of leaf tissue with antibodies against Myc (labeled with Cy3) and FLAG (labeled with AlexaFluor488). In leaves expressing *Ve1* or *Ve2* alone, the corresponding proteins were localized mainly to the cell surface, whereas the expression of both genes together resulted in the colocalization of the receptors mostly inside the cell (Fig. 3a, Supplementary Fig. 3).

Internalization of the individual Ve receptors was only observed following the introduction of the *Verticillium* ligand, an endoglucanase natriuretic plant peptide homolog^35^ (Fig. 3b, Supplementary Fig. 4). Internalization occurred within minutes, as previously reported with FLS2^9^. Modification of Ve1 ExxxLLW or Ve2 YxxF1 residues blocked the endocytosis of individual receptors in the presence of the ligand, confirming these signals are necessary for ligand-dependent endocytosis (Fig. 3c). Ligand-dependent endocytosis was still observed following the replacement of other C-terminal residues external to the ExxxLLW and YxxF1 motifs, including Ve2 YxxF2 and Ve2 PEST sequences, confirming that only ExxxLLW and YxxF1 are required. Receptor ligand-dependent endocytosis was not necessary to induce immunity and disease resistance was observed in *A. thaliana* transformed with Ve1ΔExxxLLW or Ve2ΔYxxF1 (Supplementary Fig. 5). Expression of Ve1 and Ve2 together appears to also activate endocytosis in the absence of the ligand, resulting in the constitutive recycling of the Ve heterocomplex (Fig. 3).

### Enhanced immunity in Ve1Ve2 plants involves heteromeric receptor complexes

The expression of Ve1-Myc and Ve2-FLAG simultaneously triggered the unexpected ligand-independent co-localization of both proteins (Fig. 3a, Supplementary Fig. 6). This co-localization was associated with an unexpected constitutive ligand-independent endocytosis of both proteins as recently reported for other cell-surface receptors that was not observed in individual Ve genotypes^20,21^. Evidence for the receptor internalisation rather than retention includes co-localization with the membrane marker FM-64 (Supplementary Fig. 4) and the remarkable amplified immunity and signaling (Figs. 1 and 5). One potential explanation for this observation is the formation of a heteromeric Ve1Ve2 complex, which facilitates internalization in the absence of a ligand (Supplementary Fig. 3). Heteromeric complexes have been reported for other PRRs, including FLS2, Cf9, and LeEix, but the presence of a ligand is typically required^9,11,33^. We tested this hypothesis in transient expression experiments in *N. benthamiana* by the immunoprecipitation of receptor complexes in the presence and absence of ligand using beads conjugated to polyclonal anti-Myc for the pulldown of Ve1Myc. Western blots probed with the FLAG antibody detected bands only in the plants infiltrated with both constructs, confirming the presence of a complex (Supplementary Fig. 7a). Similar experiments in plants expressing the mutated receptors lacking endocytosis signals generated the same outcome, confirming that these motifs are not required for the formation of heteromeric complexes (Supplementary Fig. 7b). Microsomal fractions revealed that the accumulation of Ve1 in the presence of ligand was reduced in the presence of Ve2 (Supplementary Fig. 7c). Increased disease resistance observed by co-expression of Ve1Ve2 was not detected in any possible Ve1Ve1 and Ve2Ve2 associations (Fig. 1b).

**Fig. 4:**
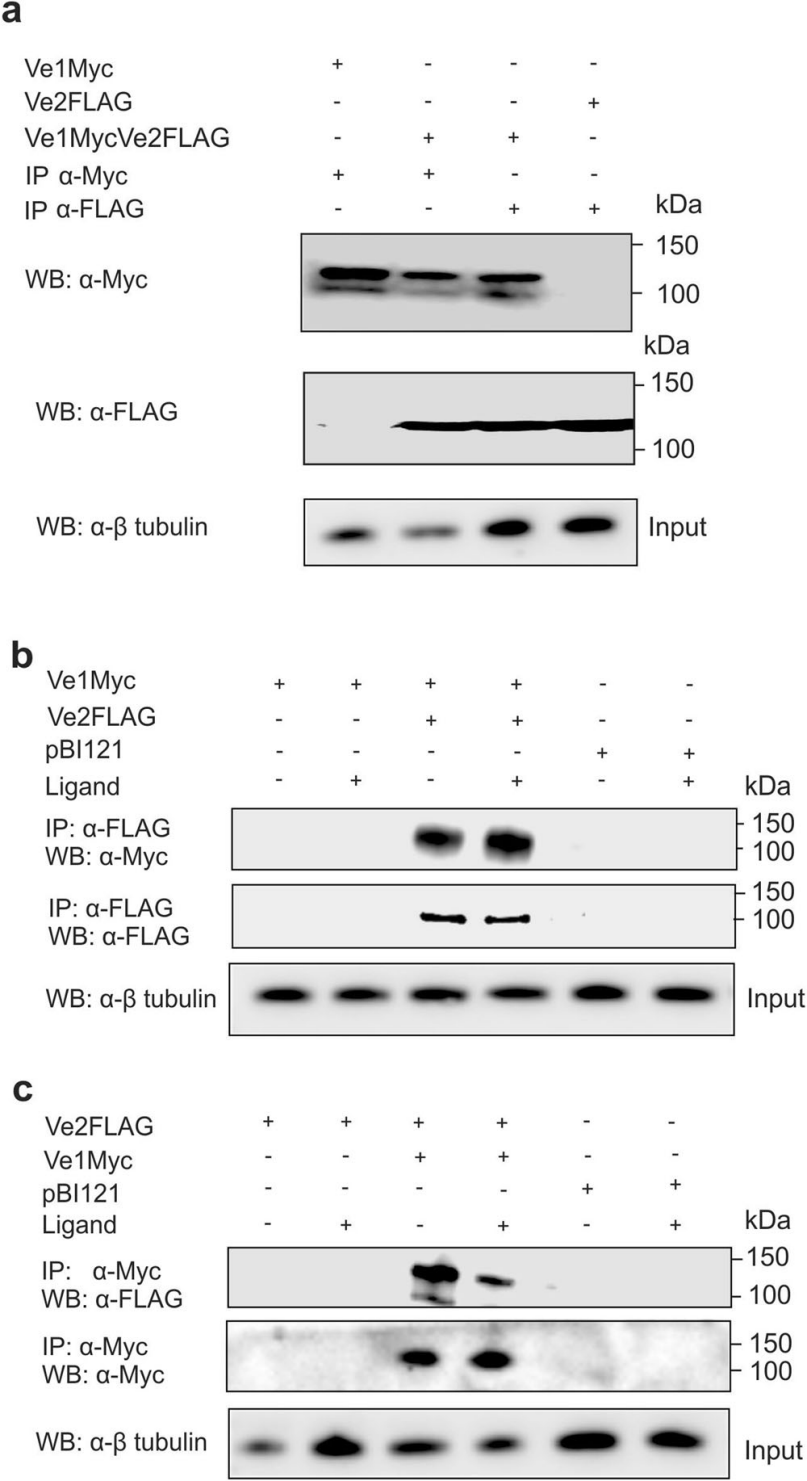
Receptor translation and heteromer composition in stably transformed *Arabidopsis thaliana* with immunoblot input shown at the top (+). **a** Western blots of proteins extracted from Ve1Myc (left lane), Ve2FLAG (right lane), and Ve1Myc + Ve2FLAG (2 center lanes) transformed plants and probed with polyclonal anti-Myc or anti-FLAG conjugated to horseradish peroxidase. Association between the receptors was confirmed by co-immunoprecipitation of Ve1 and Ve2 from the Ve1 + Ve2 co-transformed plants with beads conjugated to polyclonal anti-Myc or anti-FLAG, respectively. Input was determined by dividing samples into 3 equal fractions for the top, middle, and bottom immunoblots, using β-tubulin antisera. Antibody specificity is indicated with the reciprocal Myc and FLAG epitopes. **b** Association between the receptors was further examined by combining and incubating separate plant extracts. Co-immunoprecipitation of separately extracted Ve1 and Ve2 receptors was observed following incubation with beads conjugated to polyclonal anti-FLAG in the presence (+) or absence (-) of the ligand, and subsequent detection with receptor epitope specific Myc and FLAG antiserum but not pBI121 transformed plants. **c** Reciprocal confirmation of receptor association with co-immunoprecipitation of separately extracted Ve1 and Ve2 receptors following incubation by beads conjugated to polyclonal anti-Myc in the presence (+) or absence (-) of the ligand and subsequent detection with receptor epitope-specific antiserum. Results confirm the formation of a ligand-independent Ve1Ve2 heteromer complex.

To confirm function of tagged receptors in stably transformed plants, we generated transgenic potato (Supplementary Fig. 8) and Arabidopsis (Supplementary Fig. 5) lines expressing the two receptors individually or together. Inoculation of the plants with aggressive race 1 isolates of *Verticillium albo-atrum* confirmed disease resistance visually in the lines transformed with tagged *Ve1* and *Ve2*, as previously reported with untagged proteins^3^. Results also show similar protein expression levels for Ve1Myc and Ve2FLAG in the transformed Arabidopsis (Fig. 4) and that the endocytosis signals were not required for disease resistance (Supplementary Fig. 5). We then carried out additional immunoprecipitation experiments to test directly for receptor associations. Western blots of the *Ve1Ve2* plants probed with antibodies specific for either the Myc and FLAG tags confirmed the reciprocal immunoprecipitation of complexes containing both proteins (Fig. 4a) and the association was confirmed in vitro using beads conjugated to polyclonal anti-FLAG in the presence or absence of the ligand (added 1 h before extraction) for the pulldown of Ve2-FLAG (Fig. 4b) or beads conjugated to polyclonal anti-Myc for the pulldown of Ve1-Myc (Fig. 4c). Association with BAK1 was also observed but only in the presence of the ligand (Supplementary Fig. 9) and specificity of the Ve complex components confirmed by the absence of FLS2 in the immunoprecipitates (Supplementary Fig. 10).

### Signaling and ROS amplification by Ve1 and Ve2

**Fig. 5:**
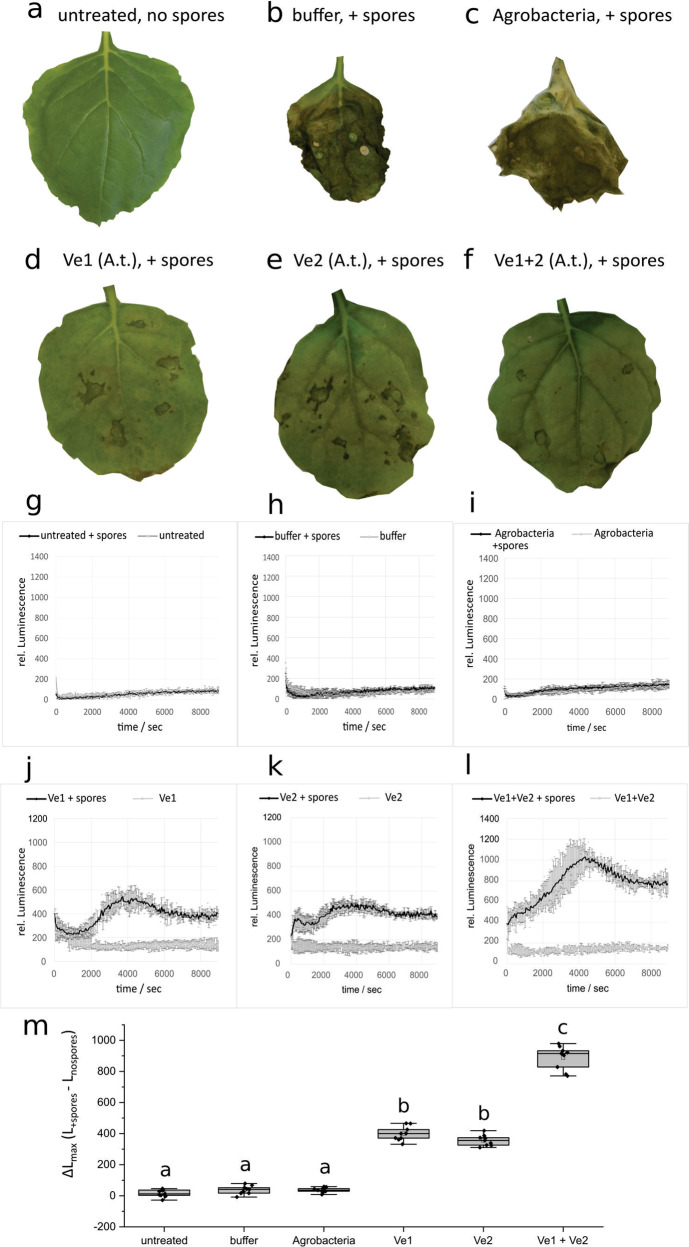
Agroinfiltration of *Nicotiana benthamiana* with *Ve* results in increased levels of reactive oxygen species (ROS). The photographs show *N. benthamiana* plants after infection with *Verticillium dahliae* (DSM 11938). No (**a**) or strong symptoms (**b**, **c**) were visible in the untreated (**a**) or spore treated (**b**, **c**) control leaves. The heterologous expression of Ve1Myc (**d**), Ve2FLAG (**e**) or the combination of the tagged receptors (**f**) was induced prior to infection, the infection symptoms appeared to be mild, especially in combination (**f**). Production of ROS triggered by signal perception (**g**–**l**). Graphs showing the rise of luminescence with *V. dahliae* elicitor in an HRP/luminol leaf disc assay that is associated with the formation of oxidative burst (*n* = 3 for each construct). In the control leaves with and without elicitor the formation of an oxidative burst is absent (**g**–**i**). The heterologous expression of Ve1Myc (**j**) and Ve2FLAG (**k**) in *N. benthamiana* leaves enable an oxidative burst that is elicited by *V. dahliae* (DSM 11938) culture. If both receptors Ve1 and Ve2 are expressed simultaneously (**l**), the recorded luminescence change significantly (*p* < 0.01) exceeds the values that were measured in the single infiltrations. The recorded maximum difference in luminescence with spore suspension and with no spores (ΔLmax) changed significantly. The values in (**m**) display ΔLmax and are marked with different letters for significant different values (*p* < 0.01).

Production of reactive oxygen species (ROS) is critical for activation and regulation of immune signaling in pathogen infections^36^. To determine the cellular response to Ve perception and signal transduction mechanism for the increased immunity, the rapid and transient burst of reactive oxygen species (ROS) was examined. In the FLS2 complex, BAK1 activates a series of trans-phosphorylation events with various intracellular associated kinases that results in downstream signaling and a rapid burst of ROS^37,38^. Recent advances show that plasma membrane-localized NADPH oxidase respiratory burst oxidase homolog (RBOH) activation and regulation occurs predominantly through N-terminal and C-terminal phosphorylation^39,40^. Transient expression of Ve1Myc and Ve2FLAG confirmed that both cell surface receptors are functional and independently capable to mediate immunity to *V. dahliae* (DSM 11938) infection, as indicated by the lack of systemic symptom development in agroinoculated *N. benthamiana* leaves (Fig. 5d, e), and the fewest disease symptoms were observed with the co-expression of both tagged receptors (Fig. 5f). As expected, control leaves showed either no (Fig. 5a) or severe (Fig. 5b, c) symptoms. Next, we verified that resistance is attributed to a signal perception that activates a ROS production persisting several hours after pathogen application (Fig. 5g–l). ROS production was observed only in leaves of *N. benthamiana* inoculated with Ve receptors and spores, and the signals were significantly enhanced when Ve1 and Ve2 were expressed simultaneously (Fig. 5j–l).

## Discussion

For many receptors, including FLS2, Cf, LeEix, PEPR1/2 and the TLRs, dimerization leads to equilibrium between the monomer and complex that constitutes part of the activation process^10,19–21,32,33^. Receptors recognize a specific ligand to induce conformational changes that activate signaling, ultimately leading to immunity and endocytosis. However, endocytosis was not required for immunity, and Ve receptors without the dileucine E/DxxxLL or tyrosine YxxΦ1 signals continued to provide effective disease resistance. Although the ligand-independent formation of Ve heteromeric complexes triggered endocytosis, this did not induce innate immunity against *V. dahliae* race 2 and infection continued unabated. The association and receptor-induced endocytosis of the Ve proteins therefore does not appear to induce disease resistance, but instead promotes race-specific signal transduction and attenuation^11^.

**Fig. 6:**
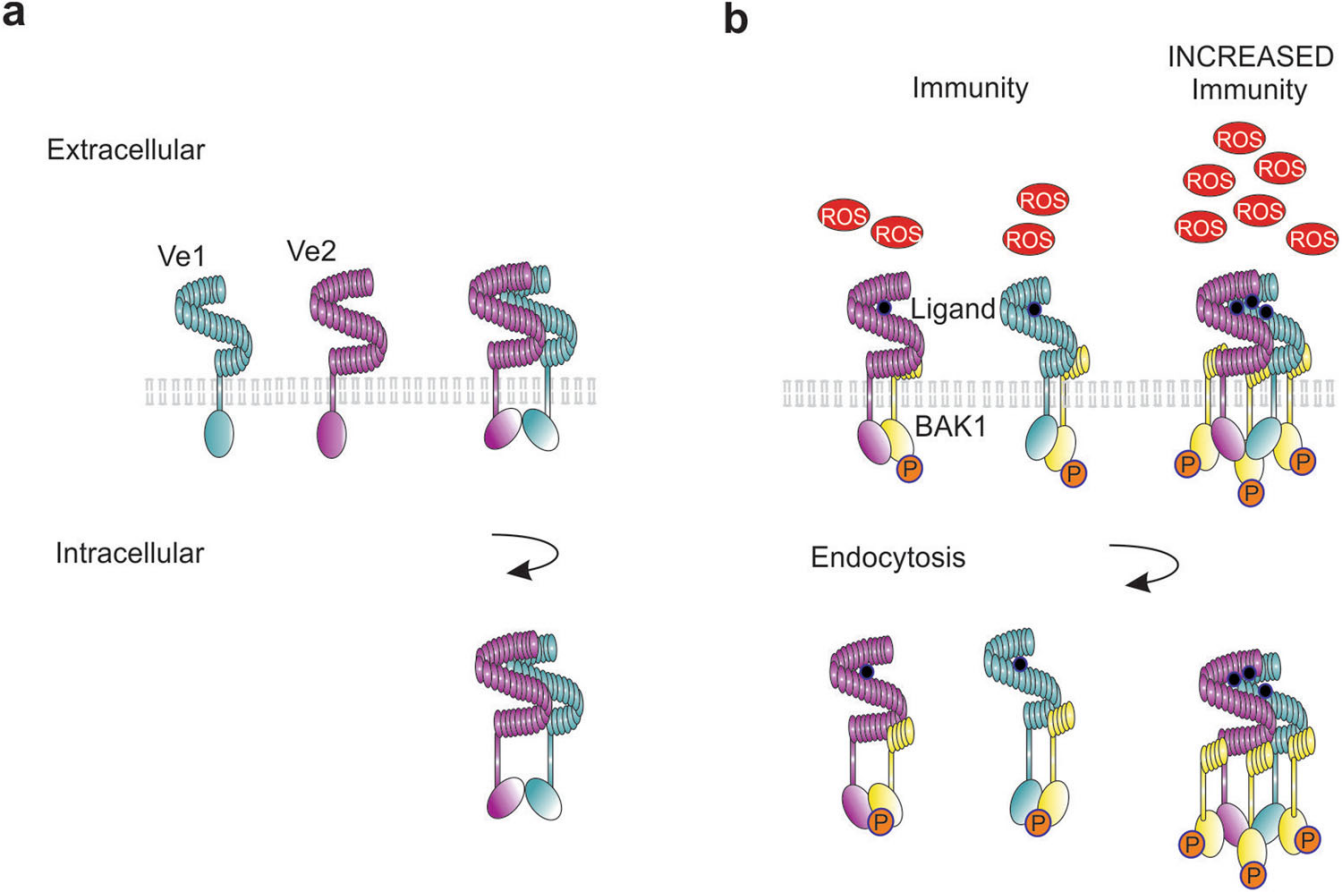
Formation of a Ve1Ve2 heteromer regulates signaling and increases disease resistance. Illustration of the Ve1 and Ve2 heterodimer showing the predicted primary interface of the receptors based on TLR complexes. Model for oligomerization places the two C-termini, transmembrane helices, and intracellular signaling domains in close spatial proximity. In the inactive state (**a**), Ve1 and Ve2 reside at the cell-surface as individual receptors and heteromerization occurs, inducing pathogen-independent endocytosis and removal. Infection by the fungal pathogen (**b**) introduces ligand recognized by Ve1 and Ve2 that induces heterocomplexes with kinases such as BAK1 and subsequently initiates pathogen induced endocytosis and signal attenuation by the activated immunity receptors. Through the formation of Ve1Ve2 heterocomplexes the immunity receptors amplify signal transduction to elevate early dying disease resistance and stimulate a reactive oxygen species (ROS) burst.

Based on the above, we propose a model in which ligand-induced activation and endocytosis of the Ve receptors rearranges the intracellular domains sufficiently to act as a scaffold for the recruitment of intracellular adaptor proteins and associated kinases, which initiate immunity (Fig. 6, Supplementary Fig. 11). Analysis indicates that the constitutively active kinase BAK1 is required for the formation of a functional immunity receptor complex^24^. Furthermore, both Ve proteins include MOD_ProDKin_1 and MOD_NEK2_1 motifs at the C-terminus that are sites for serine/threonine phosphorylation, a major cell signaling mechanism^41^. Linkage and functional assays suggest that the Ve proteins may associate with homologs in other plant species to induce immunity^42^. Although our study is limited to the analysis of two paralogous PRRs expressed in three different host species, our combined visual, molecular and functional data show that the immunity complex can be optimized by associations among homologous receptors and that signaling activity is enhanced by receptor diversity. Indeed, the increase in immunity corresponds to ROS biogenesis which is known to known to facilitate apoplastic systemic signaling that have antimicrobial properties and can target cytoplasmic components to regulate cellular activities, such as stomatal closure and callose deposition, thereby further reducing pathogen penetration^37–39^.

The tight linkage of the inverted *Ve*1 and *Ve*2 genes in most plant genotypes contributes to their coexpression, and receptor-induced endocytosis appears to be a mechanism to regulate their accumulation on the cell surface^3,11^. The linkage is likely to have arisen by gene duplication at the ancestral *Ve* locus, with the two genes then evolving to fulfil a regulatory role and refine their cell signaling activity. Related homologous Ve1 and Ve2 genes from cotton (*Gossypium barbadense*) conferred resistance to *Verticillium dahliae* in transgenic Arabidopsis (*Arabadopsis thaliana*)^43^. The transformation of isogenic Ve tomato varieties with the C-terminus of Ve2 was recently shown to significantly reduce infections by *V. dahliae* race 1, and influenced the expression of several defense-related and stress-response genes^44^. Single nucleotide polymorphisms in an allele of *Ve2* produced dysfunctional clones suggesting that small sequence changes can negatively affect signaling and disease resistance^24^. The formation of the receptor heterocomplex with specific amino acid polymorphisms thus appears to be critical in plant cell signaling, regulating endocytosis while significantly boosting immunity, and providing a strategy to increase disease resistance with optimized heterologous receptor sequences.

The activation of PRRs involves the sensing of stimuli either directly or via receptor accessory protein complexes, and signaling attenuated by removing the activated receptor from the cell surface, causing the response to be spatially and temporally regulated^10,20,21^. For example, several tomato *Cf* genes encoding plasma-membrane conferring resistance to *Cladosporium fulvum* are closely linked and confer novel resistance specificities arising through the selection of sequence diversification^10,45^. This sequence diversity and the recycling of the cell surface receptors may facilitate the recognition of different race-specific proteins and other MAMPs in a spatially and temporally dynamic fashion, resulting in an evolving immunity^30,45–47^.

There is growing evidence that transmembrane receptors have a preformed inactive dimeric structure that promotes conformational changes in receptor ectodomains and induces stable interactions between receptors^48–50^. Furthermore, the dimerization of receptors creates a new ligand-independent arrangement of the intracellular domains that provide the specificity required for adaptor binding and signal transduction. The formation of Ve complexes in the absence of a ligand (Fig. 6) resembles the FLS2 receptor kinase^33^, the stigma S-locus receptor kinase (SRK) self-incompatibility determinant in Brassicaceae^51^, and various mammalian transmembrane receptors^52,53^. As reported for the symmetrical TLR complexes, the positioning of the ectodomain restricts the transmembrane α-helices that rotate on their longitudinal axes, perpendicular to the membranes, activating the intracellular domains^50,54^. In receptor complexes that adhere to this “rotation model”, the ligand binds to the extracellular domain of the preformed dimers and induces rotation of the transmembrane domain to signal inside the cell. This pre-dimerization in the membrane and allosteric activation by ligand binding reduces the flexibility of the extracellular domain while increasing the rotation of the intracellular domain^50^. In addition to regulating immunity, the formation of Ve1/Ve2 heteromeric complexes produces a ligand-independent arrangement that activates receptor complex endocytosis. These receptor dynamics involving protein–protein associations appear to regulate signal activation and attenuation while increasing ROS signaling and immunity (Fig. 6).

Recent analysis of the HOPZ-ACTIVATED RESISTANCE 1 (ZAR1) receptor showed that it assembles into a preformed complex with resistance-related kinase 1 (RKS1) that oligomerizes into a pentameric resistosome^55^. In a similar a manner, immunoprecipitation and live cell imaging revealed that Ve1 associates with Ve2 in a ligand-independent process that forms a cell-surface immunity complex while stimulating rapid receptor-induced endocytosis. Our results also show that Ve proteins contain functional dileucine or tyrosine signals required for endocytosis. The ligand-induced and receptor-induced endocytosis indicates the importance of recycling activated Ve receptors in order to regulate signaling. Taken together, our data provide insight into cell surface receptor signaling mechanisms in plants and confirm that Ve1 and Ve2 recognize *Verticillium* MAMPs and undergo association to enhance disease resistance.

Recognition of the ligand by the Ve heteromeric complex significantly increased the strength of resistance and revealed spatial and temporal regulation resembling that observed in many human and animal transmembrane receptors^1,42,44,46,48^. Our visual, biochemical, and functional data shed light on the molecular mechanisms contributing to the activation and assembly of Ve complexes that achieve enhanced disease resistance. The successful in vitro reconstitution of the Ve immunity complex provides further opportunities for the characterization and improvement of plant cell signaling mechanisms and the tagged receptors will facilitate identification of other proteins in the immunity complex. The dynamic perception and signaling achieved by the Ve heteromeric complex advances our understanding of innate immunity and signaling, offering a strategy to improve plant perception and performance.

## Methods

### Experimental design

The overall objective of the study was to investigate the associations between Ve1 and Ve2 in three heterologous systems (*S. tuberosum*, Arabidopsis and *N. benthamiana*) by expressing the two receptors singly or together by stable transformation (potato, Arabidopsis) or transient expression (*N. benthamiana*) and analyzing the distribution of the receptor *in planta* in the presence and absence of (a) the ligand and (b) the putative endocytosis signals. Receptor tracking was achieved by integrating different epitope tags into each protein allowing visualization by immunohistochemical staining and the confirmation of associations by immunoprecipitation.

### DNA constructs and cloning

The *S. lycopersicon Ve*1 and *Ve*2 genes (accession numbers AF272367.1 and AF365929.1) were mutated using the QuickChange II site-directed mutagenesis kit (Stratagene). Complimentary oligonucleotide primers synthesized for substitution include 5’gataagcatatggggaaatgcgcagcagggttttcaagaaa3’ (*ΔVe1E/DxxLLΦ*), 5’tctcttgggcgttctggtggcatgtagtaaacttgat3’ (*ΔVe2YXXΦ1*), 5’tactggttcagttccggcggaatggaccctgggaagg3’ (*ΔVe2YXXΦ1*), and 5’gtggaacactatgcagctgcgaccgcagatgacaccga3’ (*ΔVe2PEST*). The genomic DNA sequences were transferred to pBSII (Stratagene) and introduced into *Escherichia coli* DH5α cells. Mutation targets and sites for epitope tag insertion were determined by predicting secondary structures using the ExPASy proteomic server (http://au.expasy.org/). Models were iteratively built with SWISS-MODEL and MolSoft ICM Pro software^56^. Native Ve structures were phased by molecular replacement using the MolSoft ICM superimposition tool and final structures were refined by identifying the specific residue positions and helices.

Epitopes for the triple-Myc tag (EQKLISEEDLEQKLISEEDLEQKLISEEDL) and triple-FLAG tag (DYKDHDGDYKDHDIDYKDDDDK) were incorporated into unique AscI sites within leucine-rich repeats by cloning to avoid signals in the terminal domains that may negatively impact functionality^31^, producing the variants Ve1K238Myc and Ve2K236FLAG. C-terminal substitutions in these tagged proteins were prepared to mutate the motifs Ve1∆E/DxxLLΦ, Ve2∆YxxΦ1, Ve2∆YxxΦ2, and Ve2∆PEST. Mutations were confirmed by DNA sequencing and the modified inserts were cloned into the binary vector pBI121 with a duplicated CaMV35S promoter used for stable and transient expression assays of all *Ve* constructs. Both vectors were transferred to *A. tumefaciens* strain EHA105^3^.

### Plants, transformation, and growth conditions

*Verticillium* wilt-sensitive potato plants (Desiree) were obtained from the US Potato Genbank (Sturgeon Bay, WI, USA) and were transformed as previously described^3^. Transgenic lines were selected on MS plates containing 50 μg/ml kanamycin and 250 μg/ml cefotaxime. Crosses were carried out between plants carrying individual *Ve*1 and *Ve*2 transgenes and true seed was collected from reciprocal crosses. Genotypes were selected by Southern analysis and expression of the Ve transcripts confirmed by reverse transcription of isolated RNA and amplification by PCR with sequence-specific oligonucleotides, as previously described^3,29^. All plants were grown in a glasshouse with 16-h supplemental lighting (HQI halide lights) at a constant temperature of 22/20 °C (day/night).

Arabidopsis plants were transformed by floral dip and *N. benthamiana* leaves were infiltrated tagged using *A. tumefaciens* EHA105 grown overnight at 28 °C in 5 ml of MS with antibiotics as previously described^57^. Pelleted cells were resuspended in infiltration medium (10 mM MES, pH 5.6, 10 mM MgCl_2_, and 150 μM acetosyringone) and incubated at room temperature for 90 min before resuspending the cells to the optical density 0.5 (OD_600_). Five-week-old *N. benthamiana* plants were infiltrated using a 1-ml syringe whereas Arabidopsis flowering plants were immersed in bacterial culture for 10 min. Leaves from infiltrated *N. benthamiana* plants were harvested for further processing 72 h post-inoculation. To inhibit post-transcriptional gene silencing in the transient expression experiments, *N. benthamiana* leaves were co-infiltrated with the tomato bushy stunt virus silencing suppressor P19 delivered by *A. tumefaciens* GV3101^58^.

### Immunolabeling and microscopy

Protein expression in *N. benthamiana* leaves was observed 24–72 h post-infiltration. The ligand of *V. albo-atrum* race 1 and *V. dahliae* races 1 and 2 was prepared from 3-week-old liquid cultures of each fungus grown in potato dextrose broth (PDB), centrifuged at 10,000 *g* for 2 min at 21 °C, and purified with a 45-micron sterile cellulose acetate syringe filter. Supernatants were applied during infiltration or added to detached leaves and seedlings as previously described^9,19,51^. The data presented herein represent a minimum of 20 replicates of confocal z-stack images and differential interference contrast (DIC) bright-field microscopy. The epidermis was removed and Ve1Myc and Ve2FLAG were labeled using an anti-Myc monoclonal antibody conjugated to Cy3 (Sigma-Aldrich) and an anti-FLAG monoclonal antibody conjugated to AlexaFluor 488 (Millipore) as previously described^21^. Macerated tissues were incubated for 1 h in Tris-buffered saline containing 0.1% Tween-20 (TBST), 1% nonfat dried milk and 10 μg/mL of the anti-Myc or anti-FLAG antibody. Images were captured immediately before and after the ligand was introduced and at 2-min intervals thereafter for 60 min. Images were captured using an Olympus FV1000 IX81 laser scanning confocal microscope with 30-mW argon and HeNe lasers at 488 and 568 nm, respectively.

For the analysis of protein accumulation in plants, leaf samples were snap frozen in liquid nitrogen and mechanically ground in 250 μL of extraction buffer (20 mM Tris, pH 7.5,150 mM NaCl, 1 mM EDTA, 1% Triton X-100, 0.1% SDS) supplemented with plant protease inhibitor cocktail (Sigma-Aldrich) diluted 1:100. Microsomal membranes were prepared as previously described^45^. Protein extracts were centrifuged at 10,000 *x g* for 5 min at 4 °C. The protein concentration in the supernatant was determined using the Bradford assay and the protein extracts were boiled in SDS loading buffer (120 mM Tris, pH 6.8, 50% glycerol, 6% SDS, 3 mM DTT, 1% bromophenol blue) before separation by SDS-PAGE on 10% polyacrylamide gels. Proteins were transferred to nitrocellulose using a semi-dry blotting apparatus (Bio-Rad) and the membranes were blocked in TBST containing 5% nonfat dried milk. The membranes were then incubated in TBST containing 1% nonfat dried milk and 10 μg/ml of the anti-FLAG polyclonal antibody (Cell Signaling) or anti-Myc polyclonal antibody (Sigma-Aldrich). Protein complexes were labeled with the appropriate horseradish peroxidase-conjugated secondary antibody (Santa Cruz Biotechnology) and detected using the enhanced chemiluminescence reagent (ECL), ECL-Plus or a 2:1 combination of ECL:ECL-Plus (GE Healthcare).

### Immunoprecipation and expression assays

Immunoprecipitation assays were carried out using ~ 1 g of Arabidopsis or *N. benthamiana* leaves expressing *35SVe1Myc::pBI121* + empty vector (OD_600_ = 0.7:0.7), *35SVe2Flag::pBI121* + empty vector (OD_600_ = 0.7:0.7), *35SVe1Myc::pBI121* + *35SVe2Flag::pBI121* (OD_600_ = 0.7:0.7), or *35SVe1Myc ΔVe1E/DxxLLΦ::pBI121* + *35SVe2Flag ΔVe2YXXΦ1::pBI12*1(OD600 = 0.7:0.7). Leaf samples were frozen in liquid nitrogen and ground in 3 ml cold extraction buffer (50 mM Tris-HCl pH 8, 150 mM NaCl, 10% glycerol, 1% (w/v) sodium deoxycholate) supplemented with 0.5% (w/v) Nonidet P-40 and a plant protease inhibitor cocktail (Sigma-Aldrich) as previously described^9^. Protein extracts were passed through Miracloth and centrifuged at 20,000 *x g* for 30 min at 4 °C. In addition to Ve1, Ve2, and Ve1Ve2 plant extracts, individual Ve1 and Ve2 protein extracts were combined in Eppendorfs and incubated overnight at 21 °C to further examine post-extraction association. Supernatants were incubated overnight with anti-Myc or anti-FLAG conjugated agarose beads (Sigma-Aldrich) at 4 °C and the beads were collected by centrifugation, washed three times with ice-cold extraction buffer, and once with 50 mM Tris-HCl pH 7.5 as described by the manufacturer. Proteins retained on the beads were separated by SDS-PAGE as above and analyzed by western blot using the anti-FLAG polyclonal antibody (Cell Signaling) or anti-Myc polyclonal antibody (Sigma-Aldrich) as probes. Plant extracts were divided equally into samples for immunoprecipitation and total protein Western blots with input determined by β-tubulin antisera (Sigma-Aldrich). Antisera for BAK1 and FLS2 (Agrisera) controls was used as recommended by the manufacturer. Images were captured with a Syngene G:BOX (Fisher Scientific) or X-ray film and annotated using GeneSys and CorelDraw software.

### Pathogen strains, inoculation and quantification

Aggressive isolates of *V. albo-atrum* race 1 LAva37, *V. dahliae* race 1 LAvd32 and *V. dahliae* race 2 LAvd33 were maintained in susceptible potato genotypes and propagated in PDB as previously described^3^. Conidiospores were suspended in water at a concentration of 5×10^7^ spores/mL for the inoculation of roots in 2-week-old plants. Resistance was evaluated visually on a weekly basis and the initially moist soil was allowed to dry until wilting occurred. Disease indices of plants were determined by assessing the percentage of tissue visually showing symptoms using the following scale 1 = <20%, 2 = 20 to 39%, 3 = 40 to 59%, 4 = 60 to 80%, 5 = >80%.

Pathogen titers were determined in plant tissues by double-antibody sandwich enzyme-linked immunosorbent assay (DAS-ELISA) based a monoclonal antibody specific for *V. albo-atrum* and *V. dahliae* (BioReba). Plant samples were collected from 50 plants in each treatment group in separate experiments at 3, 5 and 7 weeks post-inoculation. Samples were homogenized 1:20 in a Tris extraction buffer pH 7.4, and 200 μL of the extract transferred in duplicate to Nunc-Immuno Plates pre-coated with the monoclonal antibody^59^. The primary antibody was detected with a secondary antibody conjugated to alkaline phosphatase, which was in turn detected with the colorimetric substrate *p*-nitrophenylphosphate. The signal was quantified by spectrophotometry at an absorbance of 405 nm using an automated SpectraMAX 190 ELISA reader and SoftMAX Pro software (Molecular Devices).

### Measurement of oxidative burst detected in *Nicotiana benthamiana* leaves

The pBI-Ve1 and pBI-Ve2 constructs were transformed into *Agrobacterium tumefaciens* strain GV3101 pMP90 by electroporation. Transient expression was achieved by the simultaneous infiltration of *A. tumefaciens* strains GV3101 pMP90 (comprising Ve1/Ve2 constructs) and C58C1, carrying the pCH32 helper plasmid containing the RNA silencing suppressor protein p19 from tomato bushy stunt virus into the leaves of 4-week-old *N. benthamiana* plants^60,61^. As a negative control helper strain was infiltrated only. Afterwards, plants were cultivated in a growth chamber for 7 days under continuous light at 20 °C until infiltrated leaves were subjected to *Verticillium dahliae* (DSM 11938) or oxidative burst measurements.

Seven days post *Agrobacterium* infiltration a spore suspension with an OD_600_ of 0.5 was infiltrated into the corresponding leaves and corresponding plants were stressed by non-watering for 3 days. Then, the plants were rewatered for another 4 days and the infection was documented by photography. The oxidative burst in leave discs was measured seven days post Agrobacterium infiltration following according to the leave disc protocol^62^. A *V. dahliae* (DSM 11938) culture washed and diluted in water to an OD600 of 0.5 was utilized as elicitor. Luminol luminescence was measured on a Tecan M200 Pro plate reader. The measurements of Ve1, Ve2, and Ve1 + Ve2 were taken in parallel and confirmed by 3 repititions.

### Statistical analysis

All statistical analyses were performed using SAS software, and *P* <0.05 was taken to indicate statistical significance. Two-tailed unpaired Student’s *t* test or Tukey’s Honest Significant Difference test was conducted to compare two groups, whereas one-way analysis of variance (ANOVA) followed by Turkey-Kramer multiple comparison test was used for comparisons among multiple groups. All data are presented as the means ± SEM unless otherwise noted.

### Reporting summary

Further information on research design is available in the Nature Research Reporting Summary linked to this article.

## Supplementary information

Supplementary Information

Description of Additional Supplementary Files

Supplementary Data 1

Supplementary Data 2

Supplementary Data 3

Reporting Summary

## Data Availability

Sequence data were deposited NCBI GenBank under accession numbers AF272367 and AF365929. Source data underlying the main figures are presented in Supplementary Data 1-3 and uncropped versions of the blots are presented in Supplementary Figure 12. The data used to support the findings of this study are available from the corresponding authors.
