# Supplementary material for: Amplification of cell signaling and disease resistance by an immunity receptor Ve1Ve2 heterocomplex in plants
Source: Commun Biol. 2022 May 25;5:497. doi: 10.1038/s42003-022-03439-0 (PMC9132969; doi:10.1038/s42003-022-03439-0)
Supplement: Supplementary file 1 — Supplementary Information [file 42003_2022_3439_MOESM1_ESM.pdf]

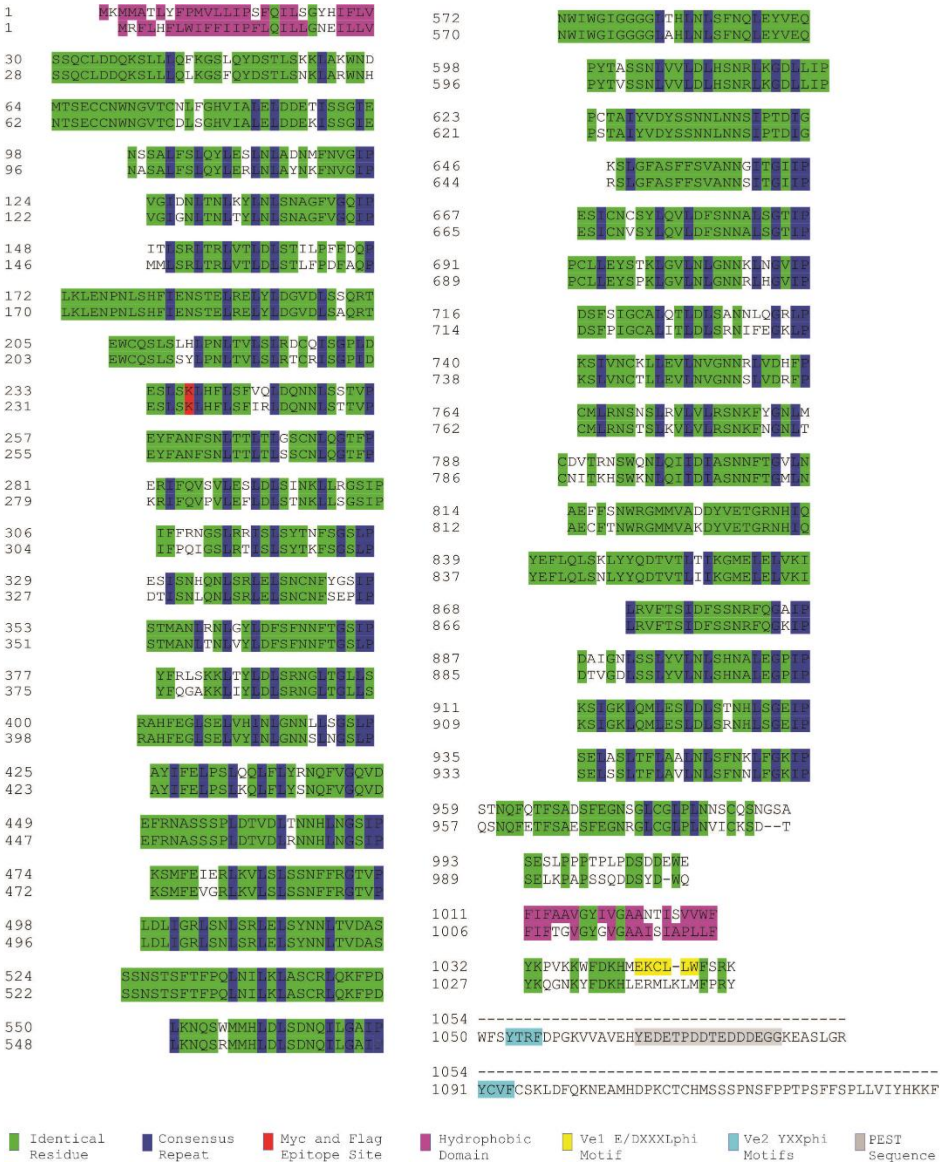

<u>*Supplementary Figure 1 alignment of Ve1 (top) and Ve2 (bottom) amino acid sequences.*</u>
Alignment shows identity, epitope insertion sites, and residues involved in endocytosis.

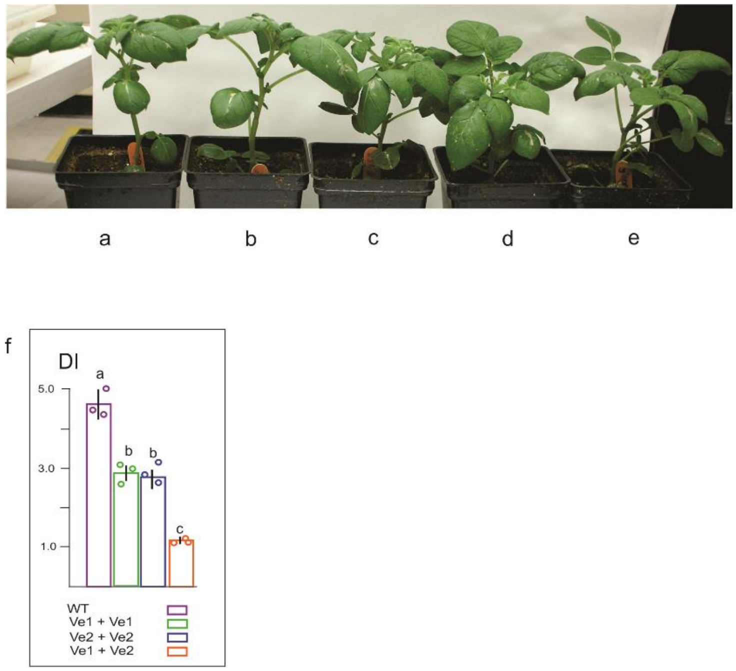

Supplementary Figure 2 plant phenotypes of potato Solanum tuberosum Desiree. (***a***) *transformed with Ve1* (***b***)*, Ve2* (***c***)*, or both immunity receptors derived from reciprocal crosses* (***d*** *and* ***e***)*. (f) Disease index (DI) for 36 plants of each genotype rated 10 weeks postinoculation with V. albo-atrum race 1 according to the percentage of tissue visually showing symptoms. Copy number of Ve was determined by sequence and Southern analysis. Means and standard error of the mean are shown indicating significant differences with one-way for ANOVA followed by Turkey-Kramer multiple comparison test ($p<0.001$) for each genotype indicated by different letters. Experiments were repeated at least three (n) times with similar results.*

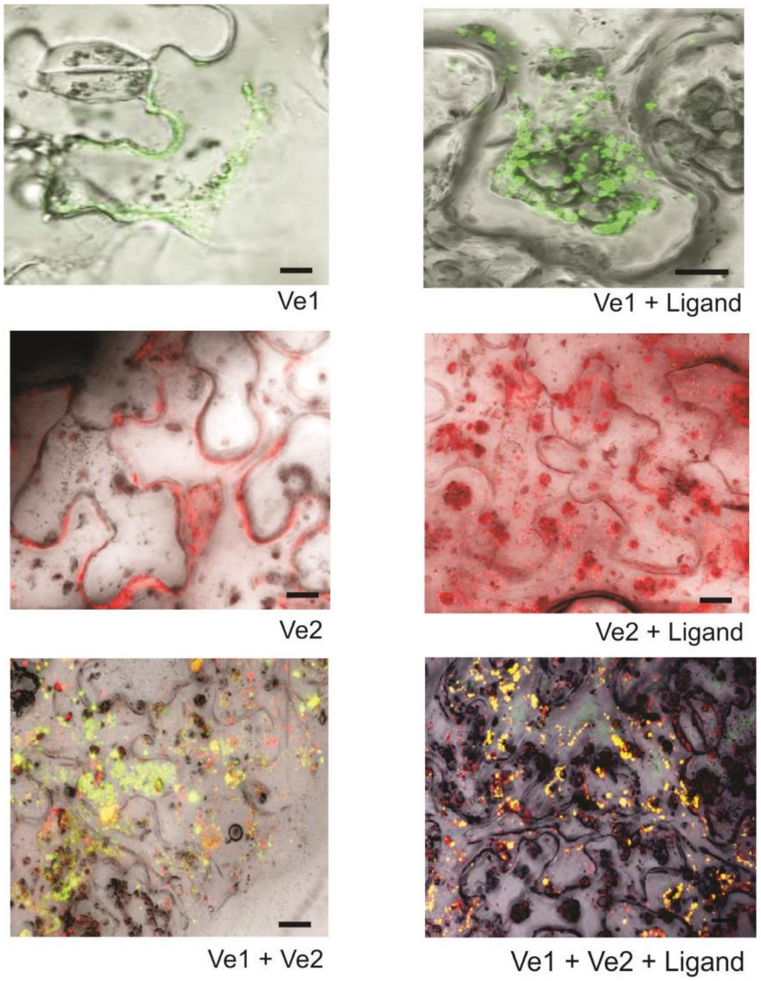

<u>Supplementary Figure 3 confocal differential interference contrast (DIC) bright field microscopy.</u> Samples incubated with fluorophore Cy3 Myc (Ve1) and ALEXA488 FLAG (Ve2) antibodies. Images show single planar sections. Size bar = 10 μm.

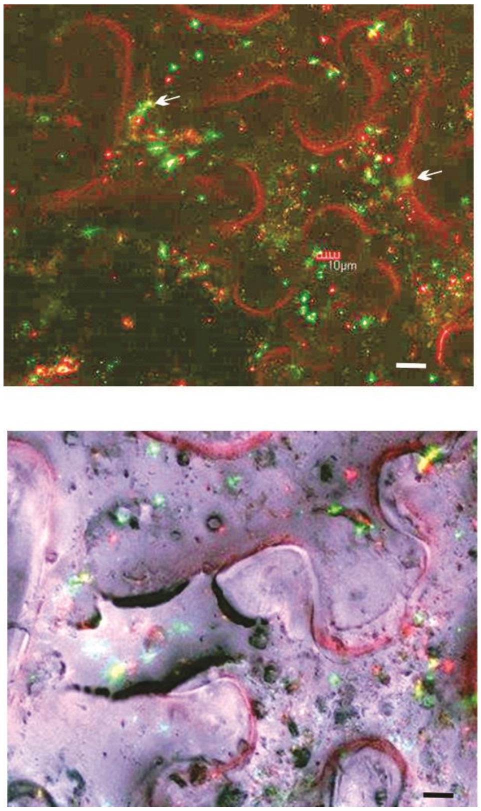

Supplementary Figure 4 confocal and differential interference contrast (DIC) bright field microscopy following sample incubation with the styryl dye FM 4-64. Sample plasma membranes fluoresce upon binding with the FM 4-64 stain following incubation with fluorophore Cy3 Myc (Ve1), ALEXA488 FLAG (Ve2) antibodies, and race 1 pathogen ligand. Arrows show co-localization of Ve1 and Ve2 receptors with the plasma membrane. Images show Z stacked sections. Size bar = 10 μm.

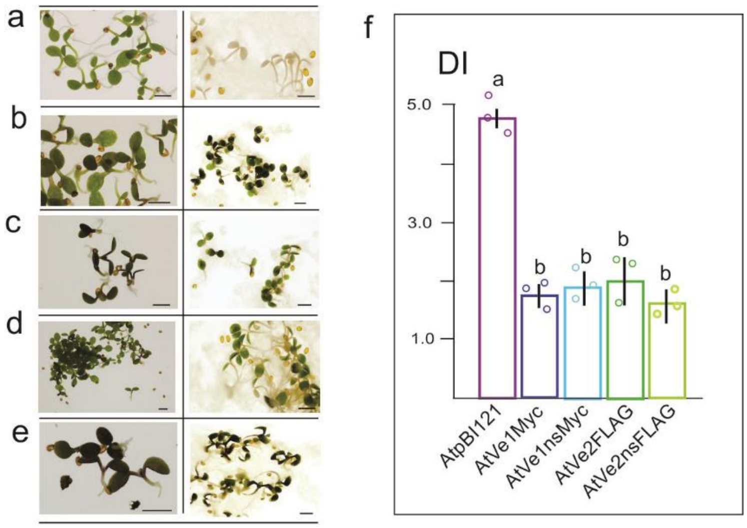

*<u>Supplementary Figure 5 disease response in transformed Arabidopsis thaliana plants.</u> Seedlings (uninoculated left) inoculated with Verticillium albo-atrum race 1 cultures (right) were chlorotic unless transformed with the tagged Ve receptors. Seedlings of stably transformed Arabidopsis thaliana containing the vector (**a**) pBI121 (At), (**b**)Ve1 (AtVe1Myc), (**c**) Ve1ΔE/DxxLLΦ (AtVe1nsMyc), (**d**) Ve2 (AtVe2FLAG), and (**e**)Ve2ΔYxxΦ1 (AtVe2nsFLAG). Seedlings show that Ve immunity functions in a heterologous plant species, endocytosis of Ve1 and Ve2 receptors is unnecessary for disease resistance, and resistance occurs with both Ve1 and Ve2 tagged receptors. Size bar = 1 mm. (f) Disease index for mature A. thaliana plants at 21 days post-inoculation of the wounded roots with race 1 of V. dahliae. Data is the average of at least 10 individual plants for each genotype from three (n) independent experiments. Significance was determined using one-way ANOVA followed by Turkey-Kramer multiple comparison test (p<0.001).*

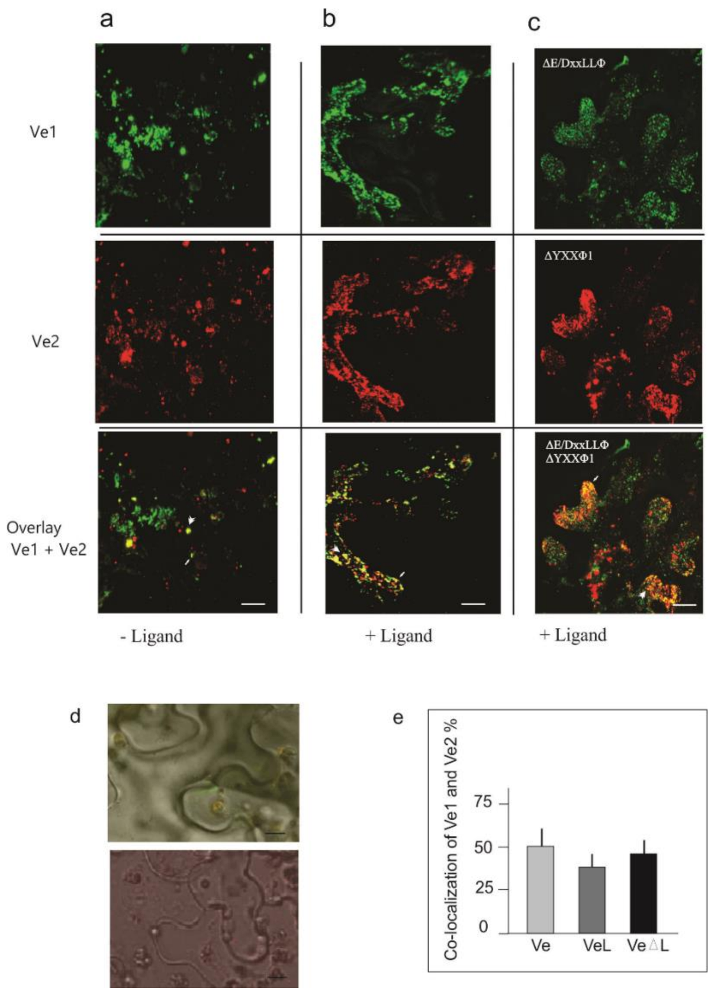

<u>Supplementary Figure 6 association overlay of the Ve1Myc and Ve2FLAG innate immunity receptor microscopic photomicrographs.</u> *Standard confocal micrographs of the agroinoculated Nicotiana benthamiana showing subcellular localization in the presence of both receptors. The panels shows Cy3 conjugated to anti-Myc (top)shown in green, AlexaFluor 488 conjugated to anti-FLAG (middle) shown in red, and overlay images of the two fluorescence signals (bottom). (**a**) Tissues without ligand (**b**) with ligand, and (**c**) receptors without endocytosis signals with ligand. Arrows indicate association at the plasma membrane and arrowheads subcellular localization. (**d**) Autofluoresence and negative confocal differential interference contrast (DIC) bright field microscopy controls. Samples incubated with fluorophore Cy3 Myc transformed with Ve2FLAG (top) or ALEXA488 FLAG transformed with Ve1Myc (bottom) antibodies. Bar = 10 μm. (**e**) Quantitative analysis of the co-localization of Ve1Myc and Ve2FLAG in the absence or presence of the ligand and endocytosis signals. Data is expressed as percentage and given as a mean and standard error of the mean (n=10) from images taken 30 min post-processing. Experiments were repeated three times with similar results.*

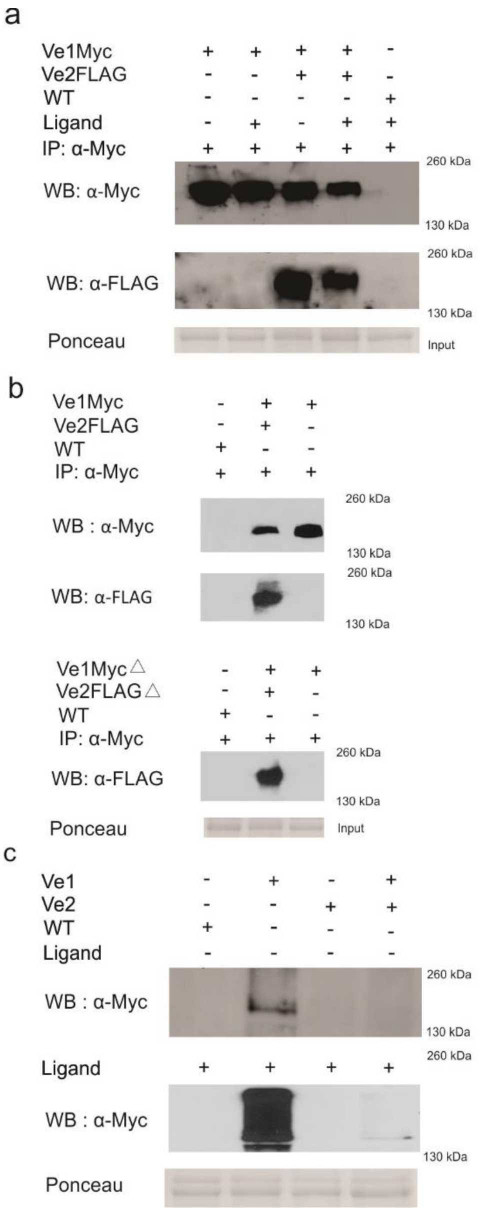

*<u>Supplementary Figure 7 Western blots of Nicotiana benthamiana leaves agroinoculated with tagged Ve receptors.</u> (**a**) Evidence for the formation of the Ve receptor heteromer in the absence and presence of pathogen ligand (10^7/ml Verticillium albo-atrum race 1 conidia). Association between the receptors was confirmed with co-immunoprecipitation of Ve1 and Ve2 receptors with beads conjugated to polyclonal anti-Myc in the presence and absence of the ligand (L) and subsequent detection with receptor specific antiserum. (**b**) Co-immunoprecipitation of the Ve1 and Ve2 (Ve1+Ve2) receptors with beads conjugated to polyclonal anti-Myc demonstrating the endocytosis signals Ve1 E/DxxLLΦ or Ve2 YxxΦ1 (Δ) are not required for the formation of the heterocomplex. (**c**) Microsomal fractions from N. benthamiana transiently agroinoculated leaves showing an increase in Ve1 receptor associated with the membrane in the presence of ligand and its reduction in the presence of the Ve2 receptor and formation of the heterocomplex.*

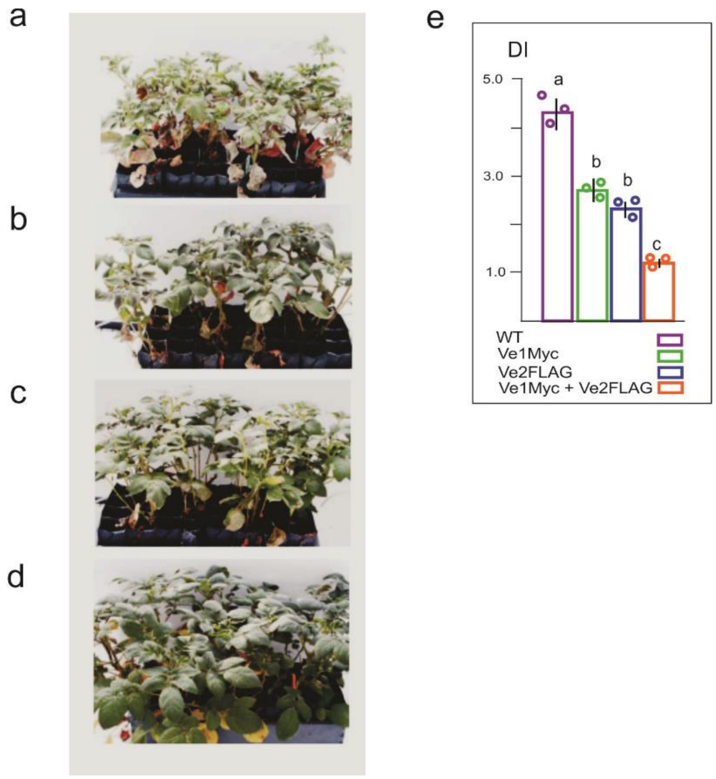

Supplementary Figure 8 disease response in Solanum tuberosum plants transformed with Ve tagged receptors. *Chlorosis, wilting, and necrosis were observed within weeks beginning in the lower leaves of untransformed plants (**a**) and progressing up from the crown causing loss of foliage. Each tray contains 36 S. tuberosum plants of the same genotype with seedlings of stably transformed Ve1Myc (**b**), Ve2FLAG (**c**), and Ve1Myc + Ve2FLAG (**d**). Seedlings were inoculated with Verticillium dahliae race 1 and show disease resistance with the immunity RPL receptors Ve1Myc and Ve2FLAG and increased resistance in the Ve1Myc + Ve2FLAG plants. (**e**) Disease index (DI) for each genotype was rated 10 weeks postinoculation according to the percentage of tissue visually showing symptoms. Mean and standard error of the mean are shown for each genotype and significant differences were determined from three (n) replicate experiments using one-way ANOVA followed by Turkey-Kramer multiple comparison test ($p<0.001$).*

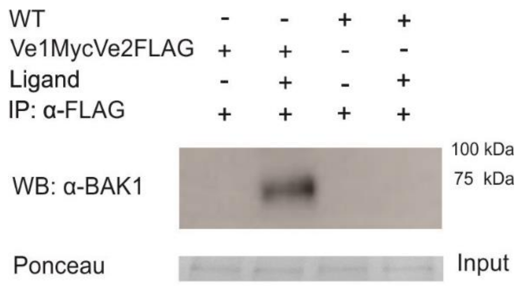

Supplementary Figure 9 immunological confirmation of receptor kinase BRASSINOSTEROID INSENSITIVE 1 ASSOCIATED KINASE 1 (BAK1) association with the Ve receptors. *Immunocprecipitation of Ve1MycVe2FLAG produced a signal with antisera specific for BAK1 only in the presence of the pathogen race 1 ligand.*

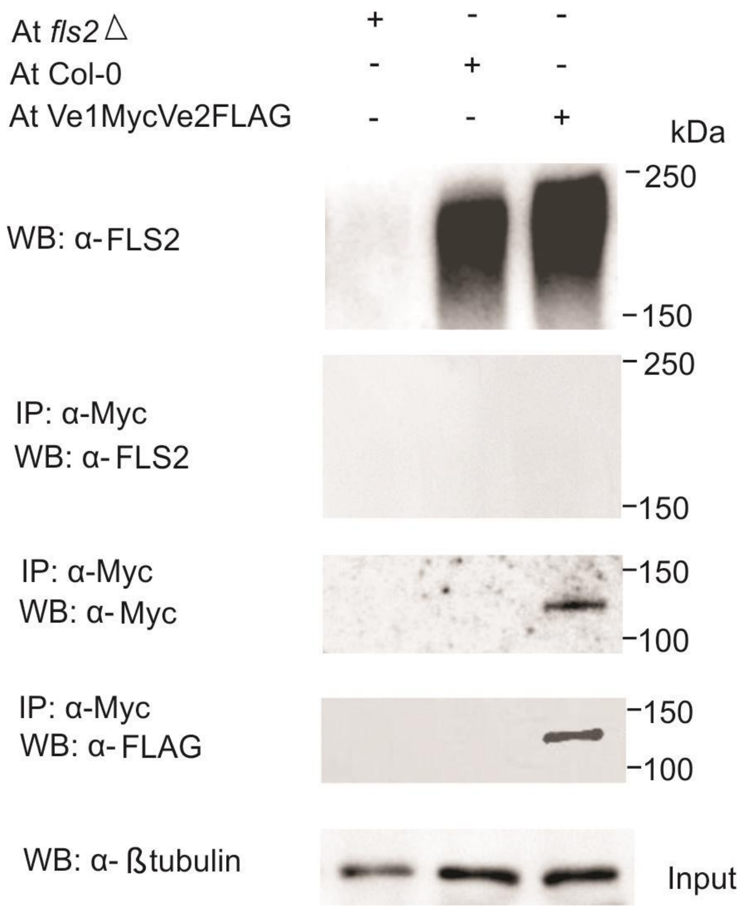

<u>Supplementary Figure 10 specificity of the Ve heterocomplex.</u> Proteins extracted from 14-day old Arabidopsis thaliana seedlings wild type Col-0, fls2 null mutant SALK093905, and the Ve1MycVe2FLAG genotype were separated by electrophoresis and blots of total protein (topblot) and Myc-agarose precipitates (middle blot) were screened with FLS2 antibody. Results show the closely related FLS2 RLK only in the total protein of A. thaliana and not in the Ve1Myc immunoprecipitates indicating specificity and removal from the Ve complex from the membrane. Input is shown in parallel screening for Ve1Myc, Ve2FLAG, and β-tubulin.

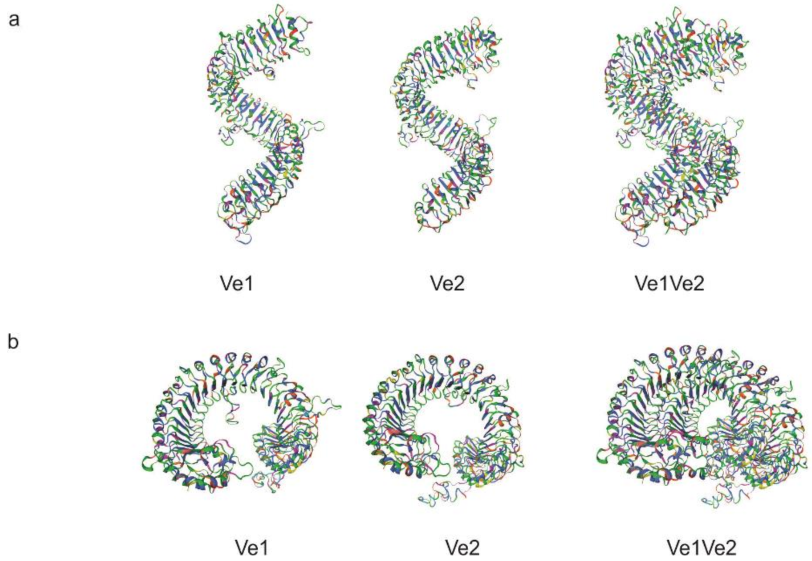

Supplementary Figure 11 illustration of the Ve1 and Ve2 ribbon heterocomplex showing the predicted secondary structure of the receptors based on LRR receptor kinase GSO1 complexes. (***a***) *Side view image showing individual Ve receptors and complex association placing the two C-termini and intracellular signaling domains in close proximity.* (***b***) *Top view of the Ve receptors and Ve1Ve2 complex that influences signaling and conferring increased disease resistance.*

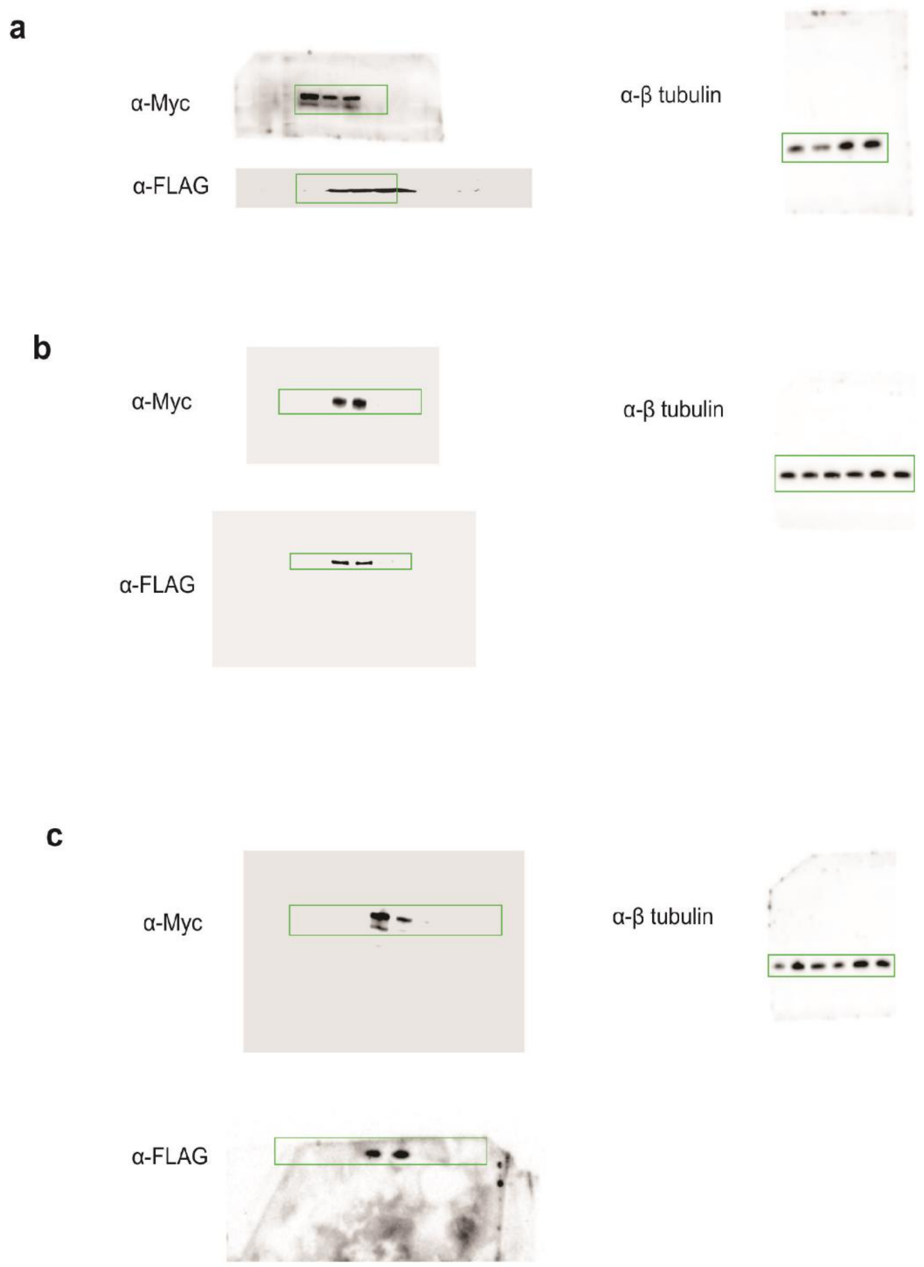

<u>Supplementary Figure 12 uncropped immunoblots.</u> *(**a**) Ve1Myc and Ve2FLAG immunoprecipitates and tubulin shown in Figure 4a. (**b**) Combined Ve1Myc and Ve2FLAG FLAG immunoprecipitates and tubulin. (**c**) Combined Ve1Myc and Ve2FLAG Myc immunoprecipitates and tubulin. Selected area of the immunoblots presented in the figures is outlined in green.*
