# Supplementary material for: Amplification of cell signaling and disease resistance by an immunity receptor Ve1Ve2 heterocomplex in plants
Source: Commun Biol. 2022 May 25;5:497. doi: 10.1038/s42003-022-03439-0 (PMC9132969; doi:10.1038/s42003-022-03439-0)
Supplement: Supplementary file 2 — Description of Additional Supplementary Files [file 42003_2022_3439_MOESM2_ESM.pdf]

Description of Additional Supplementary Files

File name: Supplementary Data 1
Description: The source data behind the graphs in Figure 1b.

File name: Supplementary Data 2
Description: The source data behind the graphs in Figure 1c and d.

File name: Supplementary Data 3
Description: The source data behind the graphs in Figure 5.
