# Supplementary material for: Amplification of cell signaling and disease resistance by an immunity receptor Ve1Ve2 heterocomplex in plants
Source: Commun Biol. 2022 May 25;5:497. doi: 10.1038/s42003-022-03439-0 (PMC9132969; doi:10.1038/s42003-022-03439-0)
Supplement: Supplementary file 6 — Reporting Summary [file 42003_2022_3439_MOESM6_ESM.pdf]

nature portfolio

Corresponding author(s): COMMSBIO-21-1982C

Last updated by author(s): 2022 04 12

Reporting Summary

Nature Portfolio wishes to improve the reproducibility of the work that we publish. This form provides structure for consistency and transparency in reporting. For further information on Nature Portfolio policies, see our Editorial Policies and the Editorial Policy Checklist.

Statistics

For all statistical analyses, confirm that the following items are present in the figure legend, table legend, main text, or Methods section.

n/a	Confirmed	
☐	☒	The exact sample size (n) for each experimental group/condition, given as a discrete number and unit of measurement
☐	☒	A statement on whether measurements were taken from distinct samples or whether the same sample was measured repeatedly
☐	☒	The statistical test(s) used AND whether they are one- or two-sided<br>*Only common tests should be described solely by name; describe more complex techniques in the Methods section.*
☒	☐	A description of all covariates tested
☒	☐	A description of any assumptions or corrections, such as tests of normality and adjustment for multiple comparisons
☐	☒	A full description of the statistical parameters including central tendency (e.g. means) or other basic estimates (e.g. regression coefficient) AND variation (e.g. standard deviation) or associated estimates of uncertainty (e.g. confidence intervals)
☐	☒	For null hypothesis testing, the test statistic (e.g. F, t, r) with confidence intervals, effect sizes, degrees of freedom and P value noted<br>*Give P values as exact values whenever suitable.*
☒	☐	For Bayesian analysis, information on the choice of priors and Markov chain Monte Carlo settings
☒	☐	For hierarchical and complex designs, identification of the appropriate level for tests and full reporting of outcomes
☒	☐	Estimates of effect sizes (e.g. Cohen's d, Pearson's r), indicating how they were calculated

Our web collection on statistics for biologists contains articles on many of the points above.

Software and code

Policy information about availability of computer code

Data collection	The softwares used for data collection in this study are described in the manuscript.
Data analysis	The softwares used for data analysis in this study are described in the manuscript.

For manuscripts utilizing custom algorithms or software that are central to the research but not yet described in published literature, software must be made available to editors and reviewers. We strongly encourage code deposition in a community repository (e.g. GitHub). See the Nature Portfolio guidelines for submitting code & software for further information.

Data

Policy information about availability of data

All manuscripts must include a data availability statement. This statement should provide the following information, where applicable:

- Accession codes, unique identifiers, or web links for publicly available datasets
- A description of any restrictions on data availability
- For clinical datasets or third party data, please ensure that the statement adheres to our policy

Sequence data were deposited NCBI GenBank under accession numbers AF272367 and AF365929. Source data underlying the main figures are presented in Supplementary Data 1-3 and uncropped versions of the blots are presented in Supplementary Figure 12. The data used to support the findings of this study are available from the corresponding authors.

Field-specific reporting

Please select the one below that is the best fit for your research. If you are not sure, read the appropriate sections before making your selection.

☒ Life sciences ☐ Behavioural & social sciences ☐ Ecological, evolutionary & environmental sciences

For a reference copy of the document with all sections, see nature.com/documents/nr-reporting-summary-flat.pdf

Life sciences study design

All studies must disclose on these points even when the disclosure is negative.

Sample size	Sample size was selected to provide reproducible results and provide statistical significance for results.
Data exclusions	No data was excluded.
Replication	All experiments were replicated with positive and negative controls and data examined by statistical analyses to identify significance.
Randomization	Pathogen isolates and gene sequences were selected to examine specificity and quantification with randomization, replication, and controls.
Blinding	Investigators were blinded in initial examination of the results prior to further investigation and analyses.

Reporting for specific materials, systems and methods

We require information from authors about some types of materials, experimental systems and methods used in many studies. Here, indicate whether each material, system or method listed is relevant to your study. If you are not sure if a list item applies to your research, read the appropriate section before selecting a response.

Materials & experimental systems

n/a	Involved in the study	
☐	☒	Antibodies
☐	☒	Eukaryotic cell lines
☒	☐	Palaeontology and archaeology
☐	☒	Animals and other organisms
☒	☐	Human research participants
☒	☐	Clinical data
☒	☐	Dual use research of concern

Methods

n/a	Involved in the study	
☒	☐	ChIP-seq
☒	☐	Flow cytometry
☒	☐	MRI-based neuroimaging

Antibodies

Antibodies used	Mouse Monoclonal Anti-c-Myc-Cy3, Sigma, clone 9E10, Catalogue number C6594.<br>Rabbit Monoclonal Anti DYKDDDDK Tag Sigma Anti-FLAG M2 epitope Alexa Fluor 488 Conjugate, Cell Signaling Technology, Catalogue #5407.<br>Rabbit polyclonal Anti-c-Myc Agarose conjugate, Sigma, Catalogue number A7470.<br>Mouse monoclonal Anti-FLAG M2 Agarose conjugate, Sigma, Catalogue number F2426.<br>Rabbit polyclonal Anti-Myc, Sigma, Catalogue number AV38156.<br>Rabbit polyclonal Anti-FLAG Sigma M2 epitope, Cell Signaling Technology, Catalogue number #2368.<br>Monoclonal anti Verticillium species DAS-ELISA, BIOREBA, Catalogue number Art-Nr: 161319.<br>BAK1 Brassinosteroid insentive 1-associated receptor kinase 1 rabbit polyclona,l Agrisera AS12 1858.<br>FLS2 Flagellin-sensitive 2 rabbit polyclonal, Agrisera AS12 1857.
Validation	Validation of each primary antibody for the species and epitope application is provided by the respective manufacturer. Further validation was conducted with controls as provided in the manuscript.

Eukaryotic cell lines

Policy information about cell lines

Cell line source(s)	Plant and microbial cultures are maintained by Agriculture and Agri-Food Canada and the University of Muenster.
Authentication	Phenotypic microscopic and molecular analyses with sequencing are routinely performed to confirm identity.
Mycoplasma contamination	No phenotypic symptoms of mycoplasma or phytoplasma was observed.

Commonly misidentified lines (See ICLAC register)	No misidentified cell lines were used.

Animals and other organisms

Policy information about studies involving animals; ARRIVE guidelines recommended for reporting animal research

Laboratory animals	Study did not involve laboratory animals.
Wild animals	Study did not involve wild animals.
Field-collected samples	Study did not involve collection of field samples.
Ethics oversight	No ethical approval was required as work was contained, did not involve plants with novel traits, and pathogens are endemic.

Note that full information on the approval of the study protocol must also be provided in the manuscript.
